# Lliga Catalana de Simulació: a post-intervention evaluation of perceived educational value following competitive clinical simulation in multidisciplinary teams

**DOI:** 10.3389/fmed.2026.1945401

**Published:** 2026-10-27

**Authors:** Munir Mohamed Mimun, Esther Jovell-Fernández, Xavier Gironès García, Aida Camps-Gómez, Ester Cañadell Yetano

**Affiliations:** 1 Simulation Committee, Consorci Sanitari de Terrassa, Hospital Universitari, Terrassa, Spain; 2 Department of Innovation and Research, Consorci Sanitari de Terrassa, Hospital Universitari, Terrassa, Spain; 3 Medical Emergency Section, Emergency Department, Hospital Clínic, Barcelona, Spain; 4 Department of Epidemiology, Consorci Sanitari de Terrassa, Hospital Universitari, Terrassa, Spain; 5 Department of Medicine, School of Medicine and Health Sciences, Universitat Internacional de Catalunya, Barcelona, Spain; 6 Chair of Simulation and Patient Safety, University of Vic – Central University of Catalonia (UVic-UCC), Manresa Campus, Manresa, Spain; 7 Research Group on Transformative Innovation and Simulation (GRITS), University of Vic – Central University of Catalonia (UVic-UCC), Manresa Campus, Manresa, Spain; 8 Department of Paediatry, Consorci Sanitari de Terrassa, Hospital Universitari, Terrassa, Spain

**Keywords:** clinical simulation, competitive simulation, cross sectional analysis, medical simulation, multicenter study, multidisciplinary team, simulation nursing education

## Abstract

**Background:**

Current medical education requires interprofessional training strategies that support the development of non-technical skills within a safe clinical learning environment. While traditional clinical simulation is widely established, the educational value of competitive and gamified environments for natural multidisciplinary teams still requires further evidence. This study aimed to characterize participants’ perceived educational experience following their participation in the Lliga Catalana de Simulació and to examine whether perceived educational outcomes and negative stress differed according to professional category and level of clinical experience.

**Methods:**

A post-intervention observational evaluation with a cross-sectional design was conducted within the context of a structured educational intervention based on progressive clinical challenges. Real multidisciplinary teams composed of nursing staff, medical residents, and attending physicians from various centers participated. Each scenario consisted of a briefing, a simulated clinical case, followed by a structured, reflective debriefing guided by expert pairs. Data collection was performed using the High-Fidelity Simulation Satisfaction Scale(ESSAF), analyzing practical utility, clinical evaluation, communication, teamwork, and stress/negative aspects.

**Results:**

Participants reported a high perception of learning and excellent overall satisfaction with the activity. The highest scores were concentrated on the practical utility of the experience and the development of communication and teamwork skills. A notable homogeneity in results was observed, with no statistically significant differences between professional profiles or correlation with years of clinical experience. Furthermore, items related to stress and negative aspects received the lowest ratings on the scale.

**Conclusion:**

The “Lliga Catalana de Simulació” was associated with high perceived educational value and satisfaction across professional groups. These findings describe participants’ post-intervention perceptions and suggest that a competitive format, when embedded within a structured instructional design and supported by psychological safety and debriefing, can be perceived as a motivating and educationally valuable experience.

## 1 Introduction

Contemporary healthcare education demands methodologies that ensure patient safety while professionals acquire and refine their competencies (1). In this context, clinical simulation has emerged as a fundamental training strategy that allows students and professionals to practice in a safe and controlled environment, replicating clinical reality interactively without placing real patients at risk, and facilitating the training of individuals, teams, and systems (2). This immersive methodology not only acts as a bridge between theoretical knowledge and practical application but has also proven to be a pillar for improving the quality and safety of healthcare (1, 3), fostering interaction with the clinical environment, including collaboration with other professionals, familiarization with resources and facilities, and integration into the processes and dynamics of the healthcare system.

Professional excellence in care requires going beyond individual clinical dexterity. Mastery of non-technical skills is imperative, as these encompass critical cognitive and interpersonal capabilities such as teamwork, communication, leadership, decision-making, and situational awareness (4, 5). Evidence demonstrates that deficiencies in these skills are a relevant contributing factor to the occurrence of clinical errors and adverse events, with a direct impact on patient safety (4, 5). In this regard, the Global Consensus on Healthcare Simulation recognizes simulation as an indispensable training environment for developing individual and collective competencies, strengthening interprofessional collaboration, optimizing team performance, and fostering the integration of professionals into increasingly complex healthcare systems (1, 4, 6).

Clinical simulation has undergone a profound evolution over the last few decades. Initially, its development was closely linked to high-acuity specialties, such as emergency medicine, anesthesiology, and critical care, where the need to train for high-risk procedures and optimize teamwork drove its adoption as a training methodology. Subsequently, scientific evidence regarding its efficacy favored its expansion into other specialties, consolidating it as a transversal methodology for the education and training of healthcare students and professionals throughout the entire educational continuum.

This growth has been accompanied by a conceptual evolution of simulation itself. If, in its early stages, the emphasis was primarily on the technology and realism of simulators, the quality of a simulation activity is now grounded in the methodological design of the learning experience. In this sense, the *Healthcare Simulation Standards of Best Practice* developed by the INACSL and the SSH, alongside the standards of the Association for Simulated Practice in Healthcare (ASPiH), have contributed to establishing an international framework of best practices based on principles such as the definition of learning objectives, briefing, psychological safety, facilitation, structured debriefing, assessment, and continuous improvement. The adoption of these standards has allowed for the homogenization of clinical simulation practice, strengthened its educational quality, and reinforced its impact on the development of professional competencies and patient safety.

The educational rationale of the present intervention is grounded in Experiential Learning Theory, which conceptualizes learning as a continuous process in which knowledge is constructed through the transformation of experience (2, 3) as well as Self-Determination Theory, which posits that intrinsic motivation is maximized when autonomy, competence, and relatedness are fostered (7). In clinical simulation, this process is particularly relevant because participants engage in a concrete clinical experience, reflect on their actions and decisions during structured debriefing, integrate this reflection with previous knowledge and subsequently apply the resulting insights to new clinical situations (2, 6). Thus, simulation should not be understood solely as exposure to a realistic clinical scenario, but as a structured experiential cycle in which action, reflection, conceptualization, and subsequent experimentation are interconnected (2–4). In the present intervention, this framework was operationalized through repeated clinical challenges followed by structured debriefing, allowing participants to transform performance and errors into opportunities for reflection and subsequent learning (1, 6). The competitive component was therefore conceived as an additional motivational context surrounding the experiential learning process rather than as the educational mechanism itself.

In parallel, healthcare education has incorporated innovative methodologies aimed at increasing motivation, engagement, and learning transfer, notably gamification (7) and competitive simulation formats. International initiatives such as SimWars® or SIMCUP (8, 9) are representative examples of this approach, which emerged initially in the fields of urgent care, emergency medicine, and critical care to train clinical teams in high-complexity scenarios. Although these formats were born with a marked competitive and demonstrative component, their evolution has been influenced by the development of international clinical simulation standards.

In recent years, international organizations such as the Society in Europe for Simulation Applied to Medicine (SESAM), through initiatives like SimUniversity, together with various national and international scientific societies, have promoted the incorporation of the methodological principles outlined in international standards into these types of activities. As a result, current competitive formats increasingly integrate essential elements such as the explicit definition of learning objectives, briefing, psychological safety, structured debriefing, and assessment (10–12), ensuring that competition acts as a means to enhance learning rather than an end in itself.

The experience detailed in this paper is framed within this evolution of clinical simulation, combining the motivating potential of competition with the systematic application of international best practice standards. This approach aims to characterize a high-quality educational experience focused on participants’ perceived development of technical and non-technical competencies, perceived teamwork-related skills, and perceived psychological safety (5, 8, 13).

Despite the growing interest in competitive simulation and gamification, a critical examination of the existing literature reveals several important limitations. Early competitive formats, such as SimWars (8), were developed primarily within emergency medicine residency education and incorporated a strong competitive and spectator-oriented component, while their evaluation was primarily qualitative and perception-based. Similarly, although SIMCUP (9) advanced the field by introducing structured OSCE-like stations and objective scoring rubrics, it focused on postgraduate medical trainees, limiting the extent to which its findings can be extrapolated to multidisciplinary clinical teams. More recent initiatives, such as Disaster Day (14), have extended competitive simulation to mass-casualty triage and demonstrated its potential for engaging large groups of learners. However, these experiences have primarily involved uniprofessional learner cohorts and have focused on short-term educational outcomes, leaving uncertainty regarding the educational experience of multidisciplinary teams with heterogeneous professional backgrounds and clinical experience.

Consequently, several questions remain insufficiently explored: (1) the extent to which competitive simulation is associated with perceived negative stress across participants from different professional backgrounds; (2) how competition is experienced within intact, natural interprofessional teams rather than homogeneous or uniprofessional learner cohorts; and (3) whether perceived educational value and satisfaction vary according to professional role and level of clinical experience.

To address these specific gaps, the present study evaluates the post-intervention educational experience reported by participants in the “Lliga Catalana de Simulació”. This intervention contributes to the literature by: (i) evaluating a multicenter competitive simulation tournament embedded within a structured experiential debriefing framework; (ii) engaging natural, intact interprofessional teams composed of nursing professionals, resident physicians, and attending physicians from multiple healthcare institutions; and (iii) using a validated psychometric instrument, the High-Fidelity Simulation Satisfaction Scale (ESSAF), to quantitatively assess perceived learning, perceived development of non-technical skills, clinical evaluation, and negative stress across professional roles and levels of clinical experience.

The primary objective of this study was to characterize participants’ perceived educational experience following participation in the Lliga Catalana de Simulació, including perceived learning, clinical applicability, teamwork-related skills, and negative stress, and to examine whether these perceptions differed according to professional category and level of clinical experience.

## 2 Materials and methods

### 2.1 Study design

A post-intervention observational evaluation with a cross-sectional design was conducted to characterize participants’ satisfaction and perceived educational experience following their participation in the “Lliga Catalana de Simulació 2026.” The initiative constituted a structured educational intervention based on simulated clinical scenarios involving healthcare teams from various hospitals. The educational intervention and its subsequent evaluation were considered as distinct components: the former consisted of the competitive simulation activity, whereas the present study evaluated participants’ perceptions collected after completion of the activity.

### 2.2 Setting and participants

The study was carried out at the Hospital Universitari Consorci Sanitari de Terrassa, in facilities specifically adapted for clinical simulation. Participants were recruited through an invitation sent to heads of studies at hospitals with previous involvement or interest in clinical simulation. Interested hospitals voluntarily enrolled in the competition and were responsible for forming their own multidisciplinary teams according to the predefined eligibility criteria. Eight teams from seven healthcare institutions participated in the competition; the Consorci Sanitari de Terrassa contributed two teams to facilitate an even number of teams for the competitive format. One initially invited hospital was unable to participate because of scheduling and availability constraints and was replaced by an additional team from the Consorci Sanitari de Terrassa.

Teams were required to include healthcare professionals from different professional categories, with at least three medical residents and mandatory representation of attending physicians and nursing staff. Team size ranged from 7 to 10 participants, depending on the availability of team members and coaches. Individual participation was voluntary, and all participants provided written informed consent. A total of 68 healthcare professionals participated in the activity and completed the ESSAF questionnaire, resulting in a 100% questionnaire response rate.

The study used a convenience sample corresponding to the professionals participating in this specific edition of the competition. No *a priori* sample size calculation or statistical power analysis was performed, as the study was conceived as an exploratory evaluation of the educational experience.

### 2.3 Intervention procedure

The educational intervention was designed as a moderate-to-high fidelity clinical simulation competition. Participants were organized into eight multidisciplinary teams, each comprising 7–10 members accompanied by one or two simulation instructors/coaches, and competed against each other in a series of clinical scenarios replicating urgent and emergent situations (Figures 1, 2).

**Figure 1:**
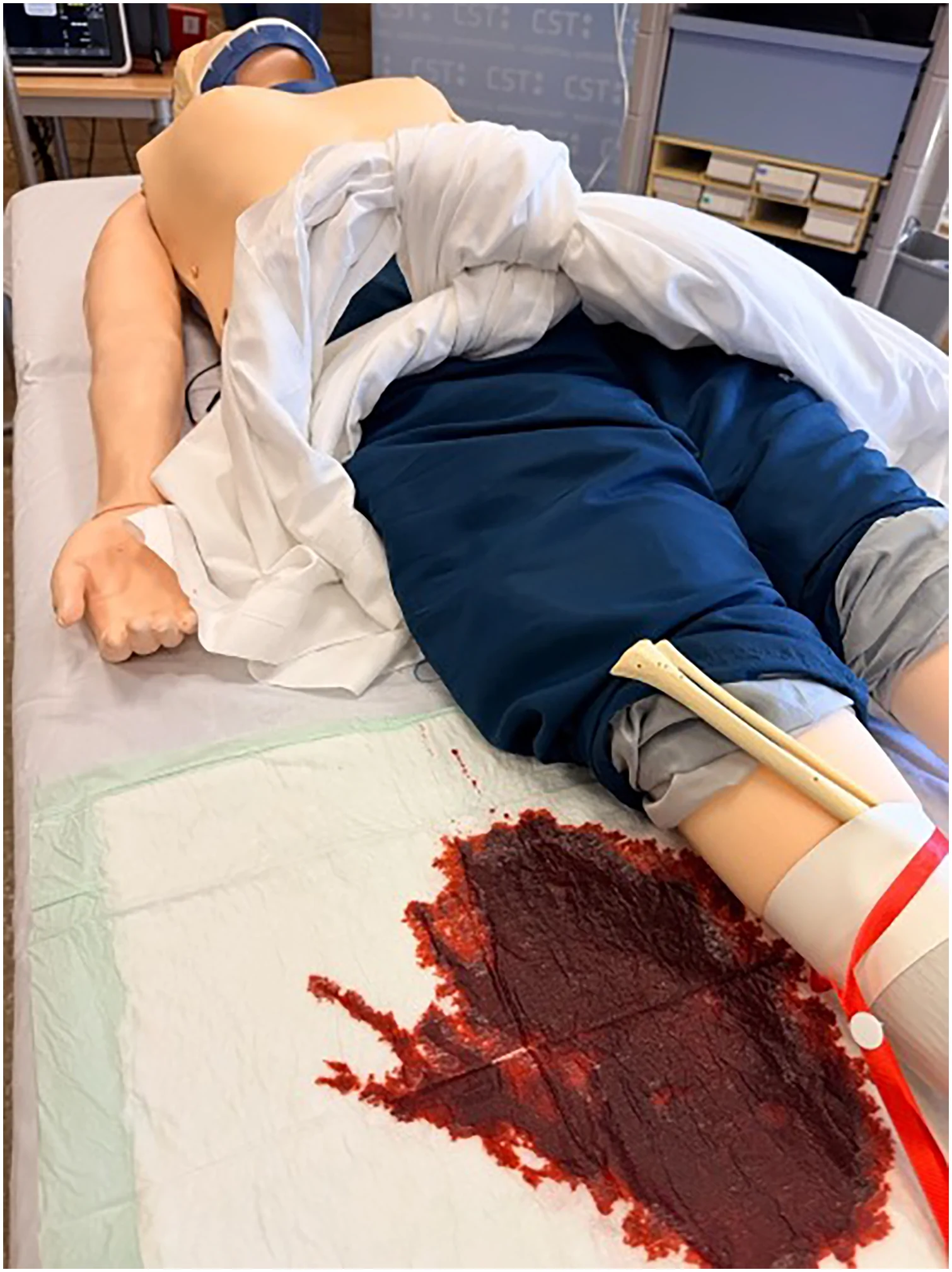
Laerdal mannequin model for polytrauma patient simulation with active hemorrhage.

**Figure 2:**
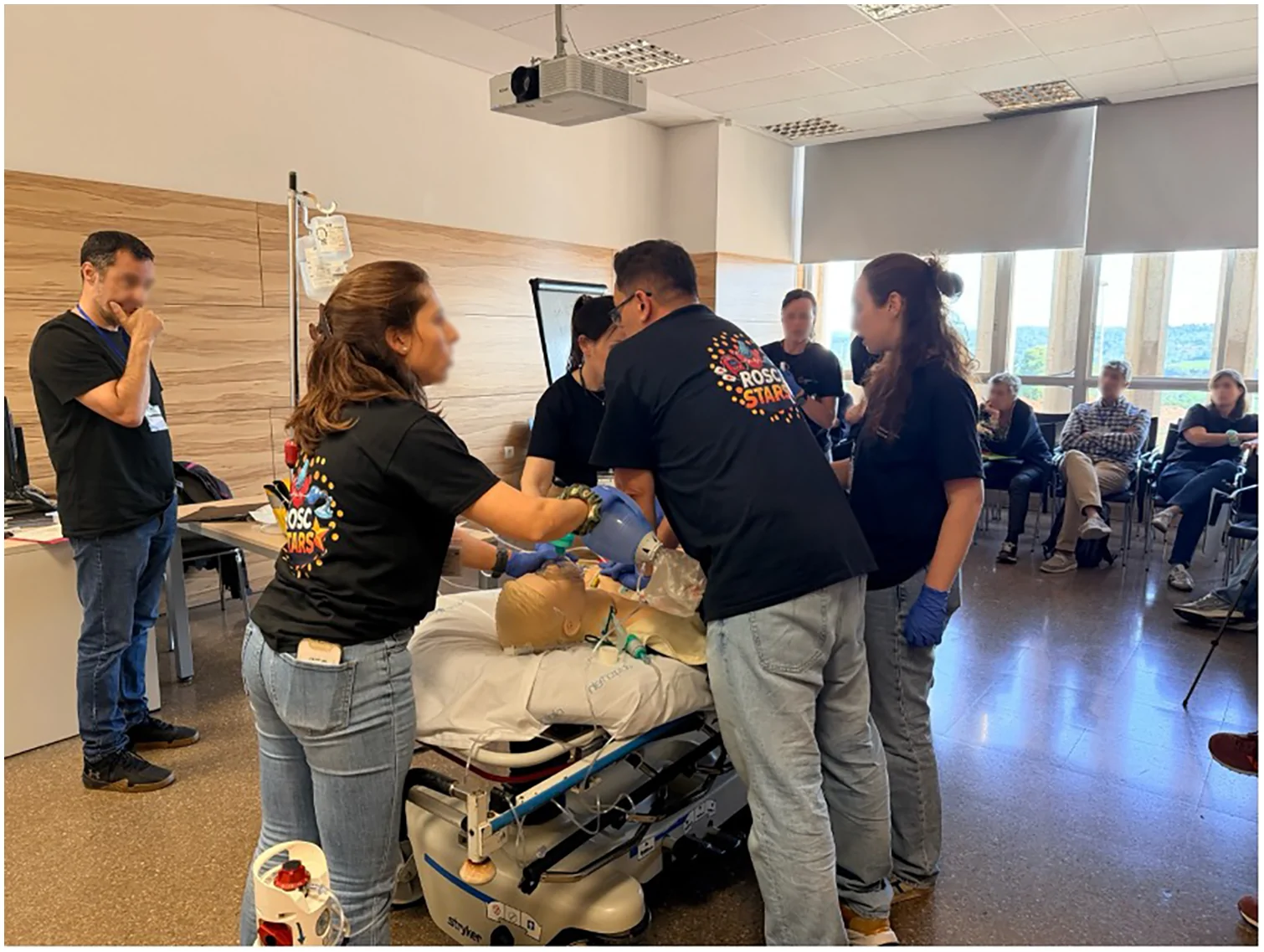
Simulation scenario of one of the teams managing a complex critically ill patient.

The competitive system included an initial group stage, in which each team performed three clinical cases against a randomly selected team. A winner was determined based on scoring rubrics, establishing a ranking according to the number of victories. Subsequently, teams advanced to a knockout stage structured into quarterfinals, semifinals, and the final. The quarterfinal represented the fourth clinical case, followed by a fifth case in the semifinal and a sixth case in the final for the teams that qualified for each successive round.

The competition was conducted over one day, with up to four simulation scenarios running simultaneously in different rooms. Each participant spent approximately 6–8 h in the activity. Each clinical case consisted of a 10–15-minute briefing, a 10–20-minute simulated scenario, and a 20–30-minute structured debriefing.

Team performance was evaluated using a rubric previously validated by a panel of judges via the Delphi method. Each clinical scenario was supervised and directed by a clinical case director. Each team was supported by one or two simulation instructors/coaches. One clinical case expert facilitated each scenario, while two judges were responsible for evaluating team performance. No additional teaching staff were involved. To guarantee quality standards in clinical simulation, the clinical cases consisted of three standardized phases:

1. **Pre-briefing/Briefing:** Before starting, an orientation was provided to familiarize participants with the simulation scenario, learning objectives, room equipment, and environment, establishing a framework of psychological safety essential for learning. The briefing lasted approximately 10–15 min.
2. **Simulated Scenario:** Real-time execution of the case, where teams were required to apply theoretical knowledge, technical skills (e.g., resuscitation or stabilization maneuvers), and non-technical skills (decision-making, leadership, situational awareness, closed-loop communication, and teamwork). Each simulated scenario lasted approximately 10–20 min.
3. **Debriefing:** Immediately following each scenario, a joint structured debriefing session was conducted by a pair of clinical case instructors and simulation experts. The debriefing lasted approximately 20–30 min. In this phase of critical reflection, participants analyzed their mental models, discussed the actions taken, and received constructive feedback to consolidate learning and apply it to real clinical practice.

### 2.4 Measures and variables

Data collection was performed using the High-Fidelity Clinical Simulation Satisfaction Scale (ESSAF) (15). The instrument consists of two main sections:

- A section for collecting sociodemographic variables (age, sex, profession, and previous experience in simulation).
- A block of 33 items evaluated using a 5-point Likert scale, with possible scores ranging from 1 to 5, where 1 corresponds to “strongly disagree” and 5 to “strongly agree.” Higher scores therefore indicate a more favorable perception of the corresponding aspect of the simulation experience, except for negatively worded items (Items 13 and 25), which were reverse-coded for the calculation of the F5 dimension.

The primary variables analyzed were the level of overall satisfaction with the simulation, perceived improvement in technical skills, perceived promotion of teamwork, adequacy of time, and the perceived contribution of the debriefing to clinical error correction.

### 2.5 Statistical analysis

Data processing and analysis were performed using descriptive and inferential statistics. The reliability and internal consistency of the ESSAF scale in the study sample were evaluated by calculating both Cronbach's alpha (α) and McDonald's omega (ω) coefficients for the overall scale and each of its five subscales. Additionally, a multiple linear regression analysis was performed to determine the predictive value of the five ESSAF dimensions on overall participant satisfaction (Item 33). For the multiple linear regression analysis, complete-case analysis was performed; participants with missing values in any variable included in the model were excluded from the regression analysis. Multicollinearity was examined using the Variance Inflation Factor (VIF).

For descriptive analysis, categorical variables (such as sex or professional category) were expressed as absolute frequencies and percentages. Continuous variables (age, years of professional experience), as well as scores obtained on the Likert scale items (1–5), were described using the mean and standard deviation (SD).

For the inferential analysis of perceived educational outcomes, the 33 items of the ESSAF scale were grouped according to the instrument’s five validated structural dimensions, allowing for the evaluation of not only satisfaction with the educational activity but also different components of perceived learning and the educational experience derived from clinical simulation:

- **F1 (Self-reflection on performance):** Items 11, 15, 17, 18, 19, 28, 29, and 31.
- **F2 (Increase in Confidence/Safety):** Items 20, 21, 22, 23, 24, and 26.
- **F3 (Relationship between theory and practice):** Items 8, 9, 10, 14, 16, 30, and 32.
- **F4 (Facilities and Realism):** Items 1, 2, 3, 4, 5, 6, and 7.
- **F5 (Stress and Negative Aspects):** Reverse items 13 and 25.

Given the ordinal nature of the Likert scale and the sample size of the subgroups, non-parametric statistical tests were employed. The Kruskal–Wallis test was used to analyze whether there were statistically significant differences in the scores of the five ESSAF dimensions based on the participants’ professional category (Nursing, Resident, and Attending Physician).

Additionally, Spearman’s correlation coefficient was used to analyze the strength and direction of the association between continuous and ordinal variables, specifically to evaluate the association between years of professional experience and perceived overall satisfaction.

All statistical calculations were performed considering values of *p* < 0.05 as statistically significant. Data processing and statistical analysis were carried out using SPSS Statistics software, version 29.0.

### 2.6 Ethical considerations

The study protocol was reviewed and approved by the Research Ethics Committee (CEIm) of the Consorci Sanitari de Terrassa, approval code 02-26-308-025. All professionals participated voluntarily and provided informed consent prior to the start of the activity. Collected data were treated with strict confidentiality using coded identifiers, ensuring compliance at all times with Regulation (EU) 2016/679 and the Organic Law 3/2018 on the Protection of Personal Data and the guarantee of digital rights.

## 3 Results

The internal consistency of the High-Fidelity Clinical Simulation Satisfaction Scale (ESSAF) was evaluated for the study population. The reliability analysis yielded a Cronbach's alpha of 0.957 and a McDonald's omega (ω) of 0.958, confirming excellent internal consistency of the instrument in this cohort. Dimension-specific reliability coefficients also demonstrated high internal consistency across subscales: F1 Self-reflection **(**α=0.939,ω=0.940**),** F2 Confidence/Safety (α=0.851,ω=0.852), F3 Theory/Practice (α=0.819,ω=0.827), and F4 Facilities/Realism (α=0.869,ω=0.872).

### 3.1 Sociodemographic characteristics of the sample

The final sample consisted of 68 participants. The mean age of the subjects was 34.3 ± 7.1 years (reported by *n* = 51), with a mean professional experience of 7.5 ± 5.4 years (reported by *n* = 52). Regarding sex distribution, 57.35% (*n* = 39) were female and 42.65% (*n* = 29) were male. Regarding professional category, of the 64 participants who reported this data, 39.06% (*n* = 25) were Residents, 37.50% (*n* = 24) were Nursing staff, and 23.44% (*n* = 15) were Attending Physicians; 4 participants did not provide this information.

### 3.2 Global descriptive analysis of the ESSAF scale

The 33 ESSAF items were analyzed descriptively to characterize participants’ perceptions across the different aspects of the simulation experience. Table 1 presents the mean scores and standard deviations for each individual item.

**Table 1:** Mean global score per item of the ESSAF scale using a 5-point Likert-type score (1–5) in the total sample. For each item, 1 indicates “strongly disagree” and 5 indicates “strongly agree”; higher scores (in bold letters in the table) indicate a more favorable perception of the corresponding aspect of the simulation experience, except for negatively worded items (Items 13 and 25).

N.°	Item	Mean	SD
**1**	The facilities and equipment were realistic.	3.69	1.03
**2**	The objectives of the cases were clear.	4.22	0.94
**3**	The cases recreated real-life situations.	4.50	0.83
**4**	The time allotted for each simulation case was adequate.	4.56	0.68
**5**	The difficulty level of the cases was appropriate for my knowledge.	4.46	0.74
**6**	I felt comfortable and respected during the sessions.	4.69	0.74
**7**	**Simulation is useful for assessing a patient's clinical situation.**	**4**.**79**	**0**.**61**
**8**	Simulation practice helps you avoid committing errors.	4.54	0.78
**9**	Simulation helped me establish priorities for action.	4.60	0.65
**10**	Simulation has improved my ability to provide care to my patients.	4.56	0.80
**11**	Simulation made me reflect on my future clinical practice.	4.59	0.80
**12**	**Simulation improves communication and teamwork skills.**	**4**.**76**	**0**.**65**
**13**	**Simulation made me feel more worried/distressed regarding the competencies that a nurse (or professional) must possess.**	**2**.**85**	**1**.**42**
**14**	Simulation is beneficial as it links theory with practice.	4.66	0.66
**15**	Simulation allowed us to plan patient care effectively.	4.41	0.78
**16**	I have improved my technical skills.	4.00	1.13
**17**	I have reinforced critical reasoning and decision-making.	4.46	0.80
**18**	Simulation helped me assess the patient's status.	4.46	0.72
**19**	This experience helped me prioritize care.	4.51	0.76
**20**	Simulation promotes self-confidence.	4.54	0.66
**21**	It allows me to improve communication with the team.	4.67	0.66
**22**	It allows me to improve communication with the family.	4.12	1.02
**23**	It allows me to improve communication with the patient.	4.13	0.96
**24**	This type of practice has increased my safety/confidence.	4.31	0.84
**25**	**I lost my composure during one of the cases**.	**2**.**59**	**1**.**30**
**26**	Interaction with simulation has improved my clinical competency.	4.35	0.75
**27**	The teacher/facilitator provided constructive feedback after each simulation session.	4.33	1.01
**28**	The analysis (debriefing) at the end of the session helped me reflect on the cases.	4.43	0.89
**29**	The analysis (debriefing) at the end of the session helped me correct errors.	4.38	0.86
**30**	I knew the theoretical part of the cases.	4.29	0.77
**31**	I learned from the errors I made during the simulation.	4.62	0.62
**32**	**Simulation has great practical utility.**	**4**.**72**	**0**.**59**
**33**	Overall, I am satisfied with the sessions.	4.56	0.80

Overall, the highest-rated items concerned the perceived usefulness of simulation for assessing a patient's clinical situation (Item 7; mean 4.79 ± 0.61), improvement in communication and teamwork skills (Item 12; mean 4.76 ± 0.65), and the practical utility of simulation (Item 32; mean 4.72 ± 0.59). Overall satisfaction with the sessions (Item 33) was also high, with a mean score of 4.56 ± 0.80.

Conversely, the lowest scores were observed for the negative items concerning loss of composure during the cases (Item 25; mean 2.59 ± 1.30) and increased worry or distress regarding professional competencies (Item 13; mean 2.85 ± 1.42).

### 3.3 Comparative analysis of ESSAF dimensions by professional category

Items were grouped into the five structural dimensions of the ESSAF scale, and their distribution was analyzed according to professional category (Nursing, Resident, and Attending Physician) (Figure 3).

**Figure 3:**
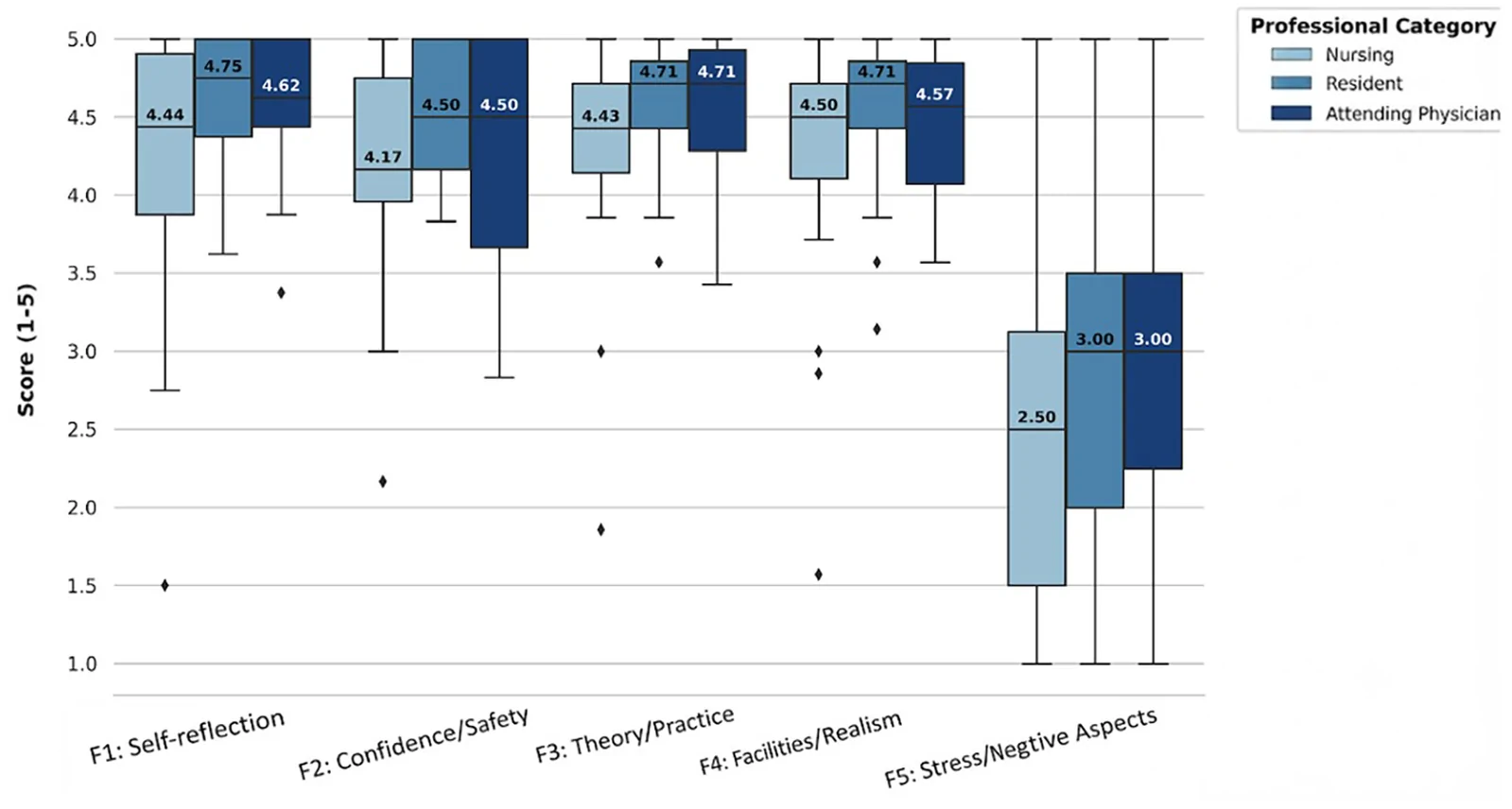
Distribution of ESSAF dimension scores by professional category. The numerical values displayed within the boxes represent the median score for each dimension and professional category. The central horizontal line within each box represents the median, while the lower and upper limits of the boxes represent the first and third quartiles (interquartile range).

The dimensions evaluated were: F1 (Self-reflection), F2 (Confidence/Safety), F3 (Theory/Practice), F4 (Facilities/Realism), and F5 (Stress/Negative Aspects). The Kruskal–Wallis test showed no statistically significant differences were observed between the three professional categories for any of the five dimensions: F1 (*p* = 0.250), F2 (*p* = 0.402), F3 (*p* = 0.182), F4 (*p* = 0.636), and F5 (*p* = 0.373).

Residents presented higher median scores and less dispersion of responses, while nursing staff showed slightly greater variability. If all professional categories are grouped together, the dimensions F3 (Theory/Practice) and F4 (Facilities/Realism) are the best-rated and show the greatest agreement among participants. In general, dimensions F1–F5 present very high scores, as the majority of the boxes are situated between 4 and 5 points on a 5-point scale, indicating a very positive global assessment of the aspects evaluated.

### 3.4 Correlation between years of experience and satisfaction

To evaluate whether previous clinical experience was associated with satisfaction with the intervention, the relationship between years of professional practice and the global satisfaction score was analyzed. The analysis indicated an absence of a statistically significant correlation (*ρ* = −0.148, *p* = 0.296), indicating no statistically significant association between years of professional experience and perceived satisfaction with the intervention.

### 3.5 Multiple linear regression analysis

To examine which dimensions of the ESSAF scale were independently associated with overall participant satisfaction (Item 33), a multiple linear regression analysis was performed using complete cases (*n* = 62). The overall regression model was statistically significant (F(5, 56) = 32.07, *p* < 0.001), accounting for 74.1% of the variance in overall satisfaction (R^2^ = 0.741, adjusted R^2^ = 0.718).

Among the five dimensions, Facilities and Realism (F4: B = 0.653, SE = 0.151, *β* = 0.519, t = 4.325, *p* < 0.001) and Self-reflection on performance (F1: B = 0.544, SE = 0.182, *β* = 0.471, t = 2.986, *p* = 0.004) were independently and positively associated with overall satisfaction. In contrast, Increase in Confidence/Safety (F2: B = −0.072, *β* = −0.059, *p* = 0.618), Relationship between theory and practice (F3: B = −0.063, *β* = −0.045, *p* = 0.735), and Stress/Negative Aspects (F5: B = −0.014, *β* = −0.018, *p* = 0.804) were not significantly associated with overall satisfaction. Multicollinearity diagnostics indicated acceptable Variance Inflation Factor values across the predictors (VIF range: 1.14–5.38).

## 4 Discussion

### 4.1 Perceived learning and methodological quality of the intervention

The main finding of this study was the high perceived educational value reported after participation in the Lliga Catalana de Simulació. The highest scores were concentrated on the practical utility of the simulation, the ability to assess the patient's clinical situation, and the perceived improvement in communication and teamwork, while overall satisfaction also reached high values. Taken together, these results indicate that the intervention was perceived not only as a satisfactory experience but as an activity with clear formative value and clinical applicability. When contrasting these findings with current literature, previous studies suggest that immersion in simulated competitive scenarios, supported by structured debriefing, may represent an effective instructional approach (15).

These findings are consistent with previous studies that used the ESSAF scale, such as those published by Guillén-Chalezquer et al. (16) and Carvajal et al. (17), who also describe high levels of satisfaction and perceived utility after clinical simulation activities. However, the present study provides a differential element compared to these investigations, as it evaluates an intervention developed in a competitive, multicenter, and interprofessional context, providing a perspective that more closely reflects the complexity of multidisciplinary clinical practice.

From a methodological perspective, these results are consistent with the paradigm shift that clinical simulation is undergoing in recent years. Traditionally, the quality of a simulation experience was primarily associated with the degree of technological fidelity of the simulator. However, the most recent international recommendations and systematic reviews agree that learning fundamentally depends on the instructional design of the experience, including the definition of objectives, the creation of a psychologically safe environment, facilitation, debriefing, and the alignment between the scenario and the competencies intended to be trained (8, 18, 19). In this regard, the high perception of learning observed in the present study may be related to the integration of these methodological elements rather than the technological level of the resources employed.

This fact is particularly relevant considering that the activity was not developed in a high-tech simulation center, but in clinical spaces adapted for training. Despite this, participants gave very high scores to the educational utility of the experience, which is consistent with what has been described by Toqan et al. (20) and Mitchell et al. (21) regarding the fact that, although high technological fidelity increases self-confidence and subjective immersion, the educational value of simulation resides primarily in the pedagogical design of the intervention and the ability to generate authentic and meaningful learning experiences, rather than in the physical fidelity of the environment.

The multiple linear regression analysis provided additional insight into the factors associated with overall satisfaction. Facilities and Realism (F4) and Self-reflection on performance (F1) were independently associated with higher overall satisfaction, whereas Confidence/Safety, Theory/Practice, and Stress/Negative Aspects were not significant independent predictors. These findings suggest that participants’ overall appraisal of the simulation may be particularly related to the perceived quality and realism of the learning environment and to the opportunity to reflect on performance. However, given the post-intervention cross-sectional design and the use of self-reported measures, these associations should be interpreted as exploratory and do not establish causal relationships or changes attributable to the intervention.

### 4.2 Transversal value, communication, and interprofessional teamwork

One of the most relevant findings of the present study was the homogeneity in the perceived educational value among different professional profiles and levels of experience. No significant differences were observed between nursing professionals, medical residents, and attending physicians in any of the ESSAF dimensions, nor was any association found between years of clinical experience and the perceived utility of the intervention. Rather than being interpreted solely as an absence of statistical differences, these results are compatible with the possibility that the activity was perceived as having transversal value by professionals with different responsibilities, disciplines, and care trajectories.

This finding is consistent with the growing consideration of clinical simulation as a strategy for continuous professional development, capable of providing benefits to both professionals in training and experienced clinicians, coinciding with the need to integrate this methodology throughout the entire continuum of the medical career to maintain professional competence (22). Likewise, this is consistent with the paradigm shift analyzed by Alblooshi et al. (23), according to which simulation has ceased to focus exclusively on the repetitive acquisition of technical skills for novices, expanding towards an educational model based on transversal, non-technical competencies applicable to experts and consolidated teams.

This interpretation acquires special interest considering that one of the aspects best valued by the participants was the perceived improvement in communication and teamwork. The high score obtained in this dimension, together with the homogeneity observed among the different professional profiles, is compatible with a perception of shared educational value beyond the individual acquisition of knowledge. Although the present study did not objectively evaluate team performance, these results are consistent with the current paradigm of clinical simulation, which places interprofessional training and the development of collective competencies as one of its main objectives (24, 25).

Team-based simulation has been described as an effective strategy for strengthening interprofessional collaboration, understanding different professional roles, and developing shared mental models, which are essential elements for safe healthcare. In this line, Farhadi et al. demonstrated that structured training through simulation was associated with improved coordination and the functioning of teams during critical situations (26), while recent systematic reviews highlight that the main value of interprofessional simulation resides in its capacity to train communication, shared leadership, and clinical coordination, rather than in the isolated learning of technical skills (1, 4, 5, 23).

The inclusion of professionals with different levels of experience and belonging to different disciplines allowed the study to examine whether perceptions of learning varied across professional groups and levels of experience. The absence of statistically significant differences suggests that participants from these groups reported a similarly positive educational experience. The study results therefore provide preliminary evidence regarding differences in perceived educational value across professionals with different levels of clinical experience. Guillén-Chalezquer et al. pointed out the need to evaluate the utility of simulation in professionals with different levels of clinical experience (16), while Vangone et al. identify the scarce characterization of the participants’ professional experience as a frequent limitation in simulation studies (1).

### 4.3 The impact of competitive gamification and the debriefing culture

Although literature warns that competitive formats can increase anxiety, favor social comparison, or even compromise learning when not designed appropriately—potentially generating cognitive overload or aversion in a significant percentage of students (27)—in the present study, the ESSAF items related to negative stress obtained the lowest scores on the entire scale. These results indicate that participants reported relatively low levels of perceived negative stress and high perceived educational value, but do not establish that competition itself was responsible for these findings.

A possible explanation for this finding resides in the methodological design of the intervention itself. Rather than promoting a competition focused exclusively on obtaining a result, the Lliga Catalana de Simulació was conceived as a training experience based on progressive challenges, where learning constituted the main objective. This approach was supported by the systematic application of principles recognized as essential for quality simulation, including a briefing aimed at establishing an environment of psychological safety, facilitation by pairs formed by a clinical expert and a simulation expert, the structured design of the scenarios, and the realization of a debriefing after each clinical case. The integration of these elements may have contributed to the favorable perception of the educational experience despite the competitive context. This aligns with previous research demonstrating that problem-based learning and overcoming challenges may enhance intrinsic motivation and problem-solving capacity (28). Thus, this type of competitive experience may provide an opportunity for professionals to practice responses to possible future critical situations, as has been evidenced in competitive simulation formats like “Disaster Day” (14).

Participants gave high scores to the utility of the debriefing for both reflection on the cases and the correction of errors. These results are consistent with works on debriefing as a tool aimed at promoting a learning culture based on reflection and continuous improvement, moving away from the search for culprits (10, 11). Similarly, Almomani et al. (29) and Shao et al. (12) show that structured debriefing models have been associated with clinical reasoning, the construction of shared mental models, and critical thinking, especially in interprofessional contexts.

From this perspective, it is probable that the high perceived learning observed in the study cannot be attributed to the competitive component in isolation, but may reflect the interaction between the challenge posed by the competition and a methodological design aligned with international best practice recommendations in clinical simulation. This approach is consistent with the current simulation paradigm, according to which the educational impact may depend substantially on the quality of the experience's design and facilitation, rather than on a single methodological element considered independently.

### 4.4 Limitations and future lines of research

Although the results are favorable, the main limitation of this study lies in the fact that the evaluation focused on satisfaction and self-perception of learning through a self-administered questionnaire. As the standardized BEME review by Issenberg et al. states, the long-term effectiveness and justification of high-fidelity simulators demand the incorporation of deliberate repetitive practice and the objective measurement of skill retention beyond the student's initial reaction (18). In future interventions, it would be interesting to incorporate validated observational tools such as the Team Emergency Assessment Measure (TEAM), which has been positioned by Gawronski et al. as the most rigorous and recommendable instrument from the psychometric point of view to adequately evaluate NTS (Non-Technical Skills) (5).

Several limitations should be considered when interpreting these findings. This was a single-event study involving a relatively small convenience sample of 68 voluntarily participating professionals, which may introduce volunteer and selection bias and limit generalizability. The absence of a comparator group and the cross-sectional post-intervention design preclude determining whether the observed perceptions were specifically attributable to the competitive format. Furthermore, outcomes were self-reported and may be influenced by social desirability, while the absence of longitudinal follow-up prevents assessment of the persistence of perceived benefits over time. The high scores observed for several ESSAF items may also suggest a potential ceiling effect. Finally, the study was conducted within a specific institutional and healthcare context, which should be considered when extrapolating the findings to other settings.

The absence of a pre-intervention assessment is also an important limitation. A pre-post design would have allowed changes in participants’ perceptions or other educational outcomes to be assessed over time and would have provided stronger evidence regarding change following the intervention. However, the present study was designed specifically as a post-intervention evaluation of the educational experience of the competition, and the ESSAF was administered after participation to assess participants’ perceived educational value and satisfaction. For future editions, a longitudinal pre-post design could be considered, incorporating appropriate baseline and post-intervention measures, ideally combining self-reported perceptions with objective assessments of individual and team performance. This approach would allow changes over time to be examined while preserving the experiential and competitive nature of the intervention.

Finally, the current critical frontier of medical simulation lies in translational science. Both Cook et al. in their extensive meta-analyses on instructional design, and Herrera-Aliaga and Estrada in their study of 21st-century innovations, make an energetic call to overcome research based purely on simulation classrooms (13, 19).

The results of this study invite a rethinking of the role of competition in clinical simulation activities. Traditionally, these types of formats have been interpreted as competitions between teams, where performance was primarily associated with obtaining a final ranking. However, the presented experience suggests a different perspective: the true educational value may not reside in the competition itself, but in the shared challenge that it generates.

From this approach, competition may act as a motivating element that may be perceived to increase the participants’ commitment, preparation, and involvement, while learning is constructed through carefully designed methodological processes, such as briefing, psychological safety, facilitation by pairs of clinical experts and simulation experts, and structured debriefing. In this context, the objective ceases to be competing against other teams to become an opportunity to learn with them.

This paradigm shift is particularly relevant when teams from different institutions participate. Beyond comparing results, these encounters can become spaces where different ways of approaching the same clinical situation can be shared, organizational models can be contrasted, decision-making can be reflected upon, and a common culture of simulation and patient safety can be built. From this perspective, future research should explore the potential of challenge-based simulation formats as inter-institutional learning communities, where success is not measured solely by the performance achieved during the scenarios, but by their capacity to favor collaborative learning, continuous improvement, and the strengthening of collective competence among professionals and healthcare organizations.

## 5 Conclusion

The results suggest that a challenge-based simulation format, developed through natural multidisciplinary teams and sustained by international best practice standards, can offer a homogeneous perceived educational experience among professionals of different disciplines and levels of experience. This finding supports the potential role of clinical simulation as a tool for continuous and interprofessional professional development, beyond training directed exclusively at novice professionals.

Likewise, the low perception of negative stress observed suggests that the competitive component, when integrated within a methodological design centered on psychological safety, expert facilitation, and structured debriefing, may be perceived as a motivating element that favors engagement and perceived learning without compromising the perceived well-being of the participants.

Finally, the results invite a rethinking of the role of competitive formats in clinical simulation. Rather than understanding competition as an end in itself, these types of initiatives could evolve towards training models based on shared challenges, where the main objective is to favor collaborative learning, exchange between teams and institutions, and the construction of a common culture of simulation and patient safety.

## Data Availability

The original contributions presented in the study are included in the article/Supplementary Material, further inquiries can be directed to the corresponding author.
